# Identification of tourists’ dynamic risk perception—the situation in Tibet

**DOI:** 10.1057/s41599-022-01335-w

**Published:** 2022-09-15

**Authors:** Yuyao Feng, Guowen Li, Xiaolei Sun, Jianping Li

**Affiliations:** 1 School of Economics and Management, University of Chinese Academy of Sciences, Beijing, China; 2 School of Management Science and Engineering, Central University of Finance and Economics, Beijing, China; 3 Institutes of Science and Development, Chinese Academy of Sciences, Beijing, China; 4 School of Public Policy and Management, University of Chinese Academy of Sciences, Beijing, China; 5 MOE Social Science Laboratory of Digital Economic Forecasts and Policy Simulation at UCAS, Beijing, China

**Keywords:** Psychology, Business and management

## Abstract

This paper proposes an identification framework for dynamic risk perception with “Questions & Answers (Q&As) + travel notes”, which newly attends to the dynamic nature of risk perception and overcomes the liabilities of traditional data collection methods, such as questionnaires and interviews, which induce high costs in data acquisition, tend to produce small sample sizes and suffer from large sample deviations. Via 2627 Q&As released by tourists before travel and 17,523 travel notes released by tourists after travel, the dynamic change in 20 identified risks before and after travel to Tibet is portrayed with the help of text mining technologies, which can automatically identify risk perception types and sentiment tendencies from massive amounts of textual data. The study finds that before travel, tourists usually underestimate risks related to safety, health and time but overestimate risks related to transportation, route selection and season. The results of the study are not only informative for destination tourism risk management and image promotion but also important for tourists to form more reasonable risk assessments.

## Introduction

As important venues for tourism activities, destinations are necessary for tourists to generate travel intentions. At the same time, destinations may be highly vulnerable to multiple risk events (Fuchs et al., 2013), and how these risks are perceived will become an important influencing factor in the process of how tourists select destinations and assess satisfaction (Chew and Jahari, 2014). Since risk perceptions are subjective judgements by the subjects, tourists’ risk perceptions of any given destination actually exist independently of the actual risk situation (Li et al., 2020; Wolff et al., 2019). From the perspective of social psychology, when destinations are geographically distant and culturally different or there are relatively large risks with inadequate information dissemination channels, tourists are prone to increase their perception of destination risks and thus make a more biased tourism decision (Mitchell, 1999). Therefore, it is very important for destination managers to correctly identify and reasonably correct the risk perceptions of tourists.

Tourists’ risk perceptions are a dynamic process (Fischhoff et al., 1983). The purposes, pathways, contents, and characteristics of destination risk perceptions vary for the same subject at different tourism stages. Risk communication theory holds that as subjects receive more information, their risk perceptions may change accordingly (Fischhoff, 1995). Therefore, for tourists, there may be a significant difference between the pretravel initial risk perceptions shaped by external factors such as risk events, media reports, and others’ reviews and the reassessed risk perceptions formed by personal experience after arriving at the destination (Tasci and Gartner, 2007). These differences may arise from risk amplification effects or tourists’ optimistic diminution of destination risks (Kapuściński and Richards, 2016; Slovic, 1987). Destination risk perceptions have an important influence on tourists’ travel behaviour, and studies have verified that there is a clear negative relationship between tourists, risk perceptions and their travel intentions as well as destination satisfaction (Karl, 2018). The overly magnified risk perceptions before travel can be a key factor in blocking tourists from pursuing travel to the destination (Wong and Yeh, 2009), while overly optimistic risk perceptions may lay hidden dangers for tourist safety and destination satisfaction (Xie et al., 2020). Therefore, it is necessary to understand the dynamic process of how tourists alter their risk perceptions of destinations and identify the differences in perceived risks before and after travel.

However, the recent research on tourist risk perception identification mainly focuses on the static perspective, which depicts the risk perceptions of tourists at the moment of data collection (Ahuja et al., 2021; Lee et al., 2015). Undeniably, these studies have contributed to understanding tourists’ risk perceptions and population differences (Yang et al., 2015). However, static risk perception studies assume that tourists’ perception of destination risks is the same at different time points, ignoring the dynamic nature of risk perceptions. There may be two difficulties in dynamically identifying tourists’ risk perceptions before and after travel. First, the data used for identification of tourist risk perceptions are mainly obtained through questionnaires and interviews. Therefore, conducting questionnaires before and after travel will double the time cost of data collection. On the other hand, the relatively small sample size may lead to the presence of sample deviation. Second, because the questionnaires generally need to preset the categories of perceived risks, they are easily limited by the designers’ knowledge, making it difficult to realise a complete, systematic identification of the perceived risks of the destination. To solve the above two problems, this paper first incorporates data from online Questions & Answers (Q&As) and travel notes released by tourists before and after travel in terms of data sources. It then takes full advantage of text mining technologies in processing massive amounts of textual data, which solves the obstacles of identifying dynamic risk perception in terms of analysis techniques.

Tibet is an attractive destination in China. Abundant species diversity, peculiar geological features and a long religious history have nurtured Tibet’s unique tourism resources, which have attracted many domestic and foreign tourists. With the opening of the railway to Tibet and the completion of airports such as Nyingchi, tourism in Tibet has entered a stage of rapid development (Su and Wall, 2009). The “China Statistical Yearbook 2020” shows that in 2019, Tibet received a total of 40.12 million domestic and foreign tourists, a year-on-year increase of 19.1%, and achieved a total tourism income of 55.93 billion yuan, which accounts for 32.94% of its economy. Tourism has become the pillar industry of Tibet’s economic development. However, the average altitude of Tibet is over 4000 metres, making it easy for tourists to suffer from physical stresses induced by altitude sickness and cardiovascular disease when travelling to Tibet (Huang et al., 2020; Labasangzhu et al., 2021). The complex terrain conditions also make Tibet mysterious and unpredictable (Bai et al., 2013; Han et al., 2017; Liu et al., 2014). The strong tourist attraction and distinctive risk characteristics make Tibet a suitable subject for studying tourists’ dynamic risk perceptions (Fuchs and Reichel, 2006). Therefore, the purpose of this paper is to identify tourists’ risk perceptions and their change processes before and after travel using Tibet as an example and to explore a general framework for the dynamic risk perception portrayal.

The contributions of this paper mainly include the following three aspects: (1) Introducing Q&As and travel notes data, and their characteristics of wide coverage, large sample size, and spontaneity can provide an effective solution to sample deviation and high time costs. (2) Realising the portrayal of dynamic risk perceptions of tourists before and after travelling to the destination based on textual data analysed with the help of text mining technologies. (3) The results can serve as supplementary information to guide tourists to form more objective pretravel risk assessments and reasonable tourism product expectations, on the other hand, it can also provide references for tourism managers to make timely adjustments to the publicity strategies for the destination image and risk management methods, thereby promoting the healthy and rapid development of the destination tourism industry.

## A review of research on identification of tourists’ risk perceptions

Tourists are both the service object and the source of benefits to the tourism industry at the destination, so the identification of their risk perceptions has always been an important branch of study to both scholars and industry managers. The existing studies on the identification of tourists’ risk perceptions can be divided into the following categories.

The first category explores the risk perceptions associated with travelling to a particular destination. Related works have identified the types and degrees of perceived risks before, during or after travel. For example, Fuchs and Reichel (2006) investigated international tourists’ perceptions of overall risks and risks in 5 specific categories (physical risk, financial risk, performance risk, socio-psychological risk, and time risk) before travelling to Israel. Adam (2015) investigated backpackers’ perceptions of six specific risks (environmental risk, political risk, financial risk, socio-psychological risk, physical risk, and expectation risk) after travelling to Ghana. Wang et al. (2019) studied tourists’ perceptions of safety risk during adventure tourism in China. Some works further summarised and categorised the identified perceived risk factors to provide more refined tourism management suggestions. For example, Simpson and Siguaw (2008) identified the risks that people care about and perceive before travel and divided them into two main categories: controllable and uncontrollable risks. Among them, controllable risks mainly include crime, the quality of tourism services and the friendliness of local residents, and uncontrollable risks include health, general psychological fears, and economic concerns.

The second category depicts tourists’ perception of a specific risk event. The main events discussed include terrorist attacks, natural disasters, and environmental risks. For example, Wolff and Larsen (2014) explored tourists’ perceptions of the risks of encountering terrorist attacks when they went to destinations where terrorist attacks occurred. Rittichainuwat et al. (2018) depicted tourists’ perceptions of natural disaster risk in areas with a history of tsunamis, proving that there is a significant positive correlation between the perceived intensity of natural disaster risk and its occurrence frequency. Becken et al. (2017) and Liang and Xue (2021) investigated the risk perceptions of air pollution by potential international tourists and domestic tourists to China. The findings show that both types of tourists have very negative perceptions of air quality at the destination, which has a significant negative impact on the destination image as well as intentions to revisit.

In the context of frequent catastrophic events, there is a growing awareness that attending high-profile events can be a high-risk behaviour, such as terrorist attacks occurred during the Boston Marathon in April 2013 and at the Stade de France in Paris in November 2015. Therefore, the third category focuses on tourists’ risk perceptions at special events. For example, Barker et al. (2003) surveyed tourists’ perceptions of crime and security risks at the 2000 America’s Cup in Auckland, New Zealand. Schroeder et al. (2013) and Walters et al. (2017) studied tourists’ risk perceptions during the 2012 and 2016 Summer Olympic Games, respectively. In addition, with the advent of the information age, tourists’ risk perceptions in the context of e-commerce have also attracted the attention of scholars. Park and Tussyadiah (2017) studied tourists’ risk perceptions when using smartphones to book travel products and identified 7 main perceived risk categories, including time risk, financial risk, performance risk, privacy/security risk, psychological risk, physical risk and equipment risk. In recent years, due to the impact of the COVID-19 epidemic, tourists’ risk perception and travel preferences have changed significantly (Li et al., 2021; Villacé-Molinero et al., 2021), making the identification of tourists’ risk perceptions during the COVID-19 pandemic a hotspot. For example, Pan et al. (2021) found that tourists had the most significant perceptions of safety, health and cleanliness risks during cruise travel.

Different from the three types of research above that use the perspective of cognitive psychology, the fourth type discusses the influence of individual characteristics and external conditions on the type and degree of tourists’ risk perceptions from a social perspective. Their studies show that gender, travel experience, travel motivation, and cultural background all have significant effects on destination risk perceptions (Reisinger and Mavondo, 2006; Yang et al., 2015). Although this type of research allows for individual differences in risk perceptions, the research samples are still cross-sectional, which depicts tourists’ risk perceptions only at the moment of data collection (Rogers, 1997).

All of the above studies are essentially static portrayals of tourists’ risk perceptions. However, risk communication theory believes that more information will bring new risk perception results (Fischhoff, 1995), that is to say, with the advancement of the travel process, the risk perception of tourists will change accordingly (Tasci and Gartner, 2007). Therefore, static identification cannot objectively reflect the full image of tourists’ risk perception. To characterise the dynamics of risk perceptions during travel, a few studies have been conducted. For example, Zimmermann et al. (2013) used a visual psychometric test to survey 314 travellers to tropical and subtropical destinations on nine health risk perceptions before and after travel. Tardivo et al. (2020) used a questionnaire and telephone interviews to examine the changes in tourist perceptions of 8 health risks before and after travel during medical tourism. Xie et al. (2020) distinguished between pretravel and post-travel risk perceptions when exploring the moderating effect of public opinion on risk perceptions and found that as tourists gained actual first-hand experience of the destination, their perceptions of the five types of risks were effectively corrected after travel.

Compared with static studies, dynamic destination risk perception studies demonstrate the variability of tourists’ risk perceptions before and after travel, which is effective for further understanding the formation and adjustment mechanism of tourists’ risk perceptions. However, the above dynamic research works mainly obtain their research data through questionnaires and interviews, which are costly in terms of data collection and are limited by relatively small samples that may lead to the existence of sample deviation. In addition, because questionnaires generally have predefined risk categories, they are easily limited by the designer’s knowledge, and it is impossible to systematically identify the full picture of the perceived risks of the destination. The above problems should be addressed and overcome in future research.

## Concept definition and theoretical foundation

### Worry, risk perceptions, and destination image

In this paper, we use Q&As and travel notes published by tourists as data for the analysis of risk perceptions before and after travel. There may be the following two concerns: (1) whether the Q&As describe worry or risk perceptions and (2) whether travel notes shape risk perceptions or destination image. In this section, we differentiate and analyse these concepts.

Risk can be divided into two categories: actual risk and perceived risk (Li et al., 2020), where actual risk can be understood as risks that exist objectively and cannot be completely eliminated (Wong and Yeh, 2009). For example, each of us faces the health risk of catching a cold, as well as the financial risk of falling interest rates every day. However, although risks exist objectively, it must be clear that the same risk means different things to different people (Li et al., 2020). Just as physically strong people usually do not worry too much about their health, they may be caught in a serious health scare during a pandemic (Ahuja et al., 2021). This process of subjective judgement of risk by different individuals can be understood as risk perceptions. The theory of risk perceptions has received extensive research attention in the field of consumer behaviour for more than 60 years, but there has never been a universally accepted definition of risk and risk perceptions. From the results of the literature review, the three elements of risk perceptions generally recognised by academics are subjective feelings, negative outcomes and objective assessment (Li et al., 2020; Sheng-Hshiung et al., 1997; Wolff et al., 2019). The product of risk perceptions can be defined as perceived risks.

The term worry is generally viewed as an important component of anxiety, which sees the vague and uncertain future as a threat. As it is often difficult for consumers to accurately report their estimates of risk probability, researchers sometimes conceptualise risk perceptions as worry and argue that there is a necessary correlation between risk perceptions and worry (Fuchs et al., 2013). Meanwhile, some researchers have questioned the correlation between them and insist that there is a fundamental difference between risk perceptions and worry. Their view is that worry is apprehension about uncertainty, which is an important factor leading to anxiety and unpleasant emotions, whereas risk perceptions are judgements about the probability of a risk occurring and potential losses. Therefore, one of them is an emotional response to uncertainty, and the other is a cognitive response to risk (Lepp et al., 2011; Rundmo, 2002; Sjöberg, 1998; Wolff and Larsen, 2014). However, risk perceptions include the perception of negative results that go beyond the subject’s own tolerance, which is itself a negative emotion. (Dowling and Staelin, 1994). Moreover, as the boundary between risk and uncertainty becomes increasingly blurred (Beck et al., 1992; Williams and Baláž, 2015), tourism risk now usually refers to the sum of risk and uncertainty (Li et al., 2020), which further weakens the differences between risk perceptions and worry.

In summary, we do not discuss further in this paper whether the Q&As describe worry or risk perceptions. On the one hand, it is difficult to make a precise distinction between them in the Q&As posted by tourists. On the other hand, this paper cares about the change in tourists’ attention to risks before and after travel. Whether tourists are worried about uncertainty or think risks will happen, this concern will become a blocking factor between tourists and the destination, so it will not interfere with the experimental results. For the same reason, although the online textual content generated by tourists, such as travel notes, is considered an important data source for shaping the destination image, the cognitive attributes involved in creating the destination image and assessing risk perceptions are the same (Perpiña et al., 2019), and tourists’ risk perceptions are also an important part of how a destination image is shaped (Xie et al., 2020). Thus, whether these post hoc assessments are positive (e.g., that it is safe) or negative (e.g., that there are severe security risks), they can be considered categorically as part of the concern for security risk for the purpose of this study.

Therefore, in this paper, we adopt a broader definition to describe risk perceptions, that is, tourists’ perceptions and worries about destination attributes, services, and risks that influence tourist travel decisions and satisfaction.

### Destination risk perceptions pre- and post-travel

Tourism is a risk-sensitive industry. Tourism activities are highly vulnerable to a variety of external factors, such as climatic conditions, unfriendly locals, health threats and language barriers. (Fuchs et al., 2013), and the perception and assessment of these risks is an important factor in shaping tourist travel decisions and satisfaction.

**Fig. 1:**
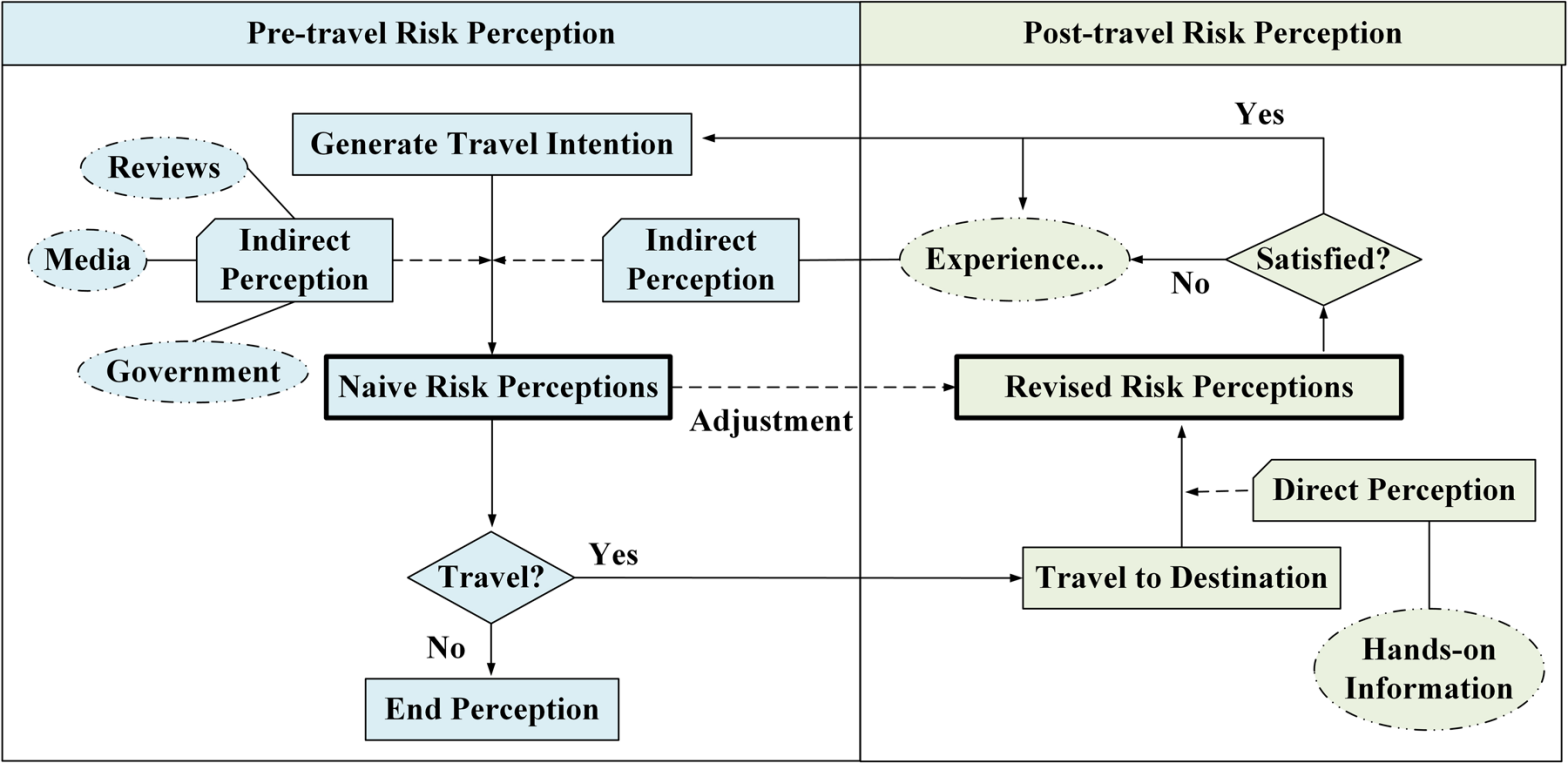
The formation mechanism of destination risk perceptions before and after travel. A description of the differences in information acquisition ways and the of risk perception purpose before and after travel.

As shown in Fig. 1, the risk perception process for a particular destination begins when tourists have an intention to travel (Fig. 1). Before travelling, since tourists have not yet arrived at the destination, their risk perceptions are indirect, mostly coming from secondary information such as government propaganda, news reports, and others’ comments (Xie et al., 2020). In addition, prior knowledge, such as travel experience, is also important supplementary information. Through the absorption of this information, tourists will form initial risk perceptions, which are named “naive risk perceptions” in this paper, and they are an important influencing factor in travel decisions. When naive risk perceptions are beyond a tourist’s acceptable range, they become a key factor that separates tourists from the destination. If the naive risk perceptions are within the acceptable range, then tourists will travel to the destination, assuming no other possible influencing factors.

After arriving at the destination, tourists have the opportunity to interact with the place in person. During the visit, they will obtain first-hand information about the destination risks through direct perception (Gartner, 1994). On the basis of the initial risk perceptions, the reassessments, which are named “revised risk perceptions” in this paper, are formed through continuous modification. Revised risk perceptions are important factors affecting tourist destination satisfaction, and higher satisfaction will positively impact motivations to revisit (Jang and Feng, 2007; Kim et al., 2009). These perceptions will serve as prior knowledge to provide information for the formation of risk perceptions before the next trip.

Given that the perceived pathways (indirect perceptions or direct perceptions) and the perceived purposes (travel decision or destination satisfaction) of tourists’ risk perceptions before and after travel are different, we believe that there may be significant differences in perceived risks between these two times. By comparison, we can describe the dynamic adjustment process of tourists’ risk perceptions after travel and identify risk factors that are magnified or underestimated by tourists before travel. On the one hand, this knowledge can help tourism managers carry out destination image promotion, reduce tourists’ perceptions of exaggerated risks, and remind tourists to raise their vigilance for underestimated risks. On the other hand, it can also provide a reference for destination risk management. For example, managers should focus on risk factors that are perceived to be aggravated after travel to improve destination satisfaction.

## Methodology and data

**Fig. 2:**
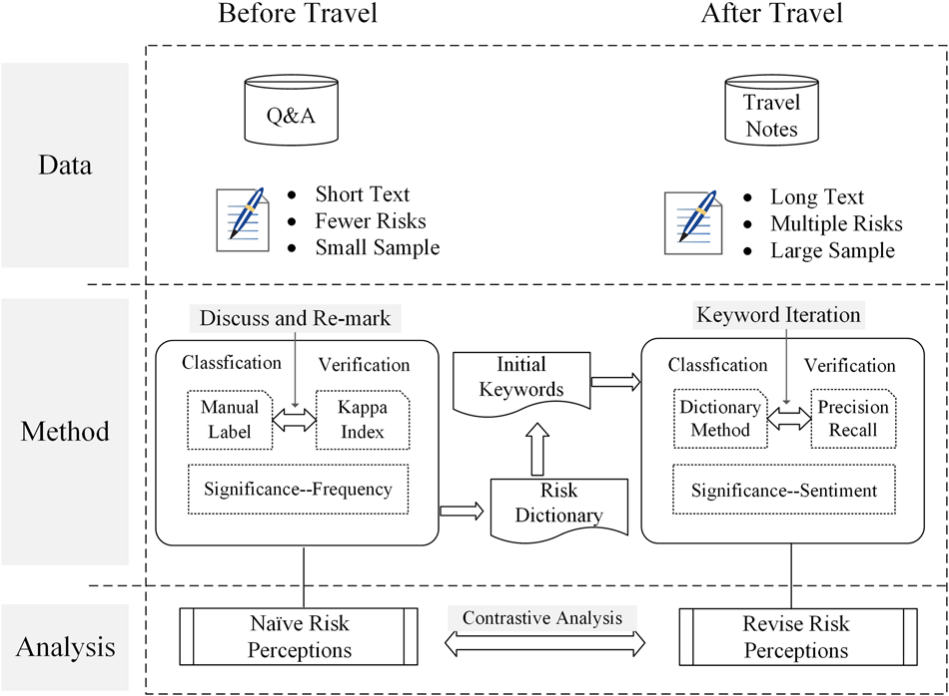
Experimental framework. It demonstrates the experimental data, methods and analysis process.

Based on the essential characteristics of the research data in this study, specific text mining technologies, including manual labelling, the dictionary method and sentiment analysis, are used to explore the risk perceptions of tourists. Figure 2 shows the empirical research framework, which is divided into three progressive levels of data, methods and analysis (Fig. 2).

This study uses Q&As and travel notes as sources of risk perceptions before and after travel, respectively. Q&As have a short text length, involve fewer risk factors, and hold a relatively small number of samples. They are suitable for use with manual labelling methods to determine the type of risk perceptions. In contrast, the length of travel notes is long, the types of risks involved are more complex and scattered, and the number of samples is larger, so it is suitable to use dictionary methods to portray risk perceptions. Furthermore, various sentiment analysis methods are used to analyse the sentiment tendency of tourists to evaluate destinations to judge the post-travel perceived importance of different risks. Finally, through comparative analysis, we can further depict the change and correction process after travelling to the destination to identify the key risk factors that are underestimated or exaggerated. The remainder of this section will describe the specifics of the text mining technologies employed.

### Naive risk perception assessment

We collected a total of 3117 Q&As from the website of Ctrip (the largest online travel service company in China) with Tibet as the destination to characterise the pretravel risk perceptions of tourists. The Q&As are questions about the uncertainties and risks of a destination posted by tourists after they generate travel intention, which can reflect naive risk perceptions of the destination to a certain extent. For example, “How to prevent altitude sickness” indicates that tourists perceived strong health risks, and “I heard that there are many mudslides, is it very dangerous?” clearly points out tourists’ concerns about the safety risks brought by natural disasters. Through data cleaning, we retained 2627 records directly related to travel to Tibet. After inspection, the earliest recorded sample can be traced back to August 2008, and the latest sample was released in April 2021. Table 1 shows the specific data screening process (Table 1).

**Table 1:** Pretravel sample selection process.

Sample selection	Count
All Q&As	3117
Not questions specific to Tibet, such as “Where is the grassland more fun?”	(122)
Without specifying the risk types, such as “What should I pay attention to when going to Tibet?”	(167)
Repeated questions from the same user in a short period of time	(201)
Final samples	2627

To identify the types of risks perceived by tourists before travelling, based on the characteristics of short text, smaller sample size, and fewer risks in each record of the Q&As, two research members with backgrounds in tourism risk research are selected to manually label the type of risk involved in each Q&A record, and the experimental process is divided into the following main stages.

1. Before the experiment, we introduced the background and purpose of the experiment to the participants in detail.
2. The experimental group previewed all the Q&A samples, discussed the labelling attributes (single- or multilabel) and predefined the risk labelling categories. By browsing through the Q&A samples, we found that each Q&A record usually involved questions about multiple risks, so the risk labelling of the Q&As belonged to a multilabel classification problem. Based on this premise, the experimental group discusses several main risk categories, including accommodation, transportation, routes, safety, religious culture and so on. Then, a unified tag word was set for each category so that comments such as “I heard that there are many mudslides, is it very dangerous?” will be labelled “safety risk”.
3. Next, we organised participants to read the Q&As one by one and mark the category of risk perception. Additionally, the predefined risk list was dynamically adjusted based on the annotated results at an intermediate point. Although there are some automatic labelling methods, these methods often fail when the research focuses on a specific area requiring expertise (Li et al., 2020). In such cases, manual labelling methods usually guarantee high accuracy (Bao and Datta, 2014; Wei et al., 2019). As shown in Fig. 3, to ensure the consistency of the labelling results, during the experiment, the first experimenter labelled all the Q&A samples and counted the number of risks labelled for each record (see Fig. 3). When labelling, if there are risks not previously defined in the previous step, the first participant is responsible for updating the list of predefined risk lists. Next, the second participant needs to label each Q&A record with a given number of risk labels (the number of risks labelled by the first participant), using the updated risk lists as a reference. For example, if the first participant labelled the first record with “traffic risk”, “safety risk”, and “health risk”, then the second participant will be asked to mark three risk labels for the same record. Similarly, for risks that have not been predefined in advance, the second participant also needs to update the risk lists.

**Fig. 3:**
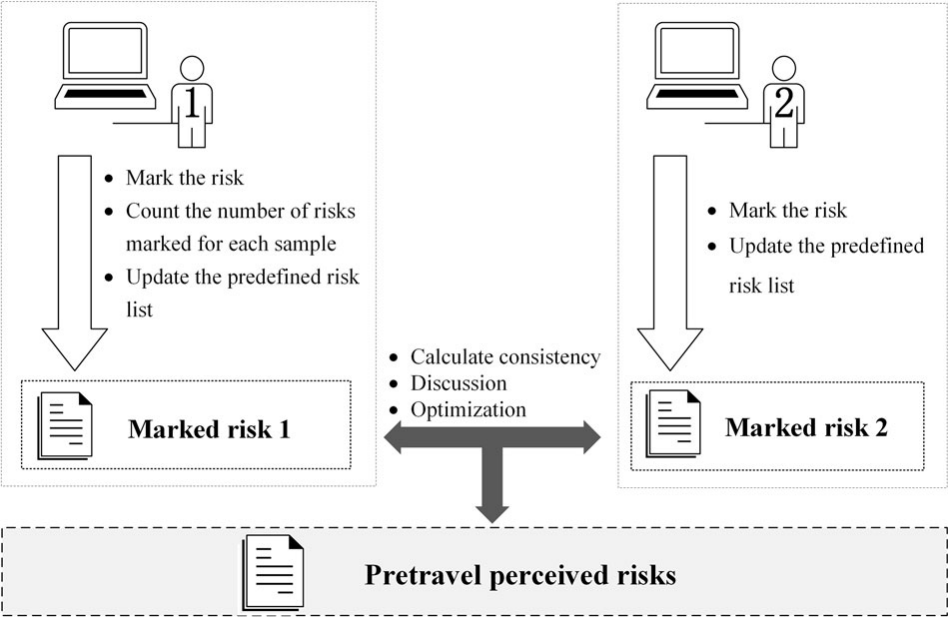
Risk label process. It visually demonstrates the manual labelling process of pretravel risk perception results.
4. To ensure the consistency of the labelling of the results of different participants, we carefully calculated the kappa index of the labels of different participants. We then discussed the divergent label results to determine the final risk perception.
5. Finally, we obtained the final risk label results and calculated the frequency of occurrence to reflect the perceived importance of different risks.

### Revised risk perception assessment

The end of a trip does not mean the end of the tourist perceptions. Tourists will share their perceptions of the destination images and risks through personal memories, small talk with friends and relatives, online reviews and travel notes. To characterise the perceived risks of tourists after travel, we crawled 17,523 travel notes about Tibet from the Ctrip website. Two specific text mining technologies are used to process these travel notes. First, considering that a single travel note is often long and records various risk perceptions at the same time, coupled with the large amount of sample data, it is difficult to identify the type of risk perception solely by manual labelling. Therefore, we identify tourist risk perceptions by constructing thematic dictionaries related to different risk perception types.

**Fig. 4:**
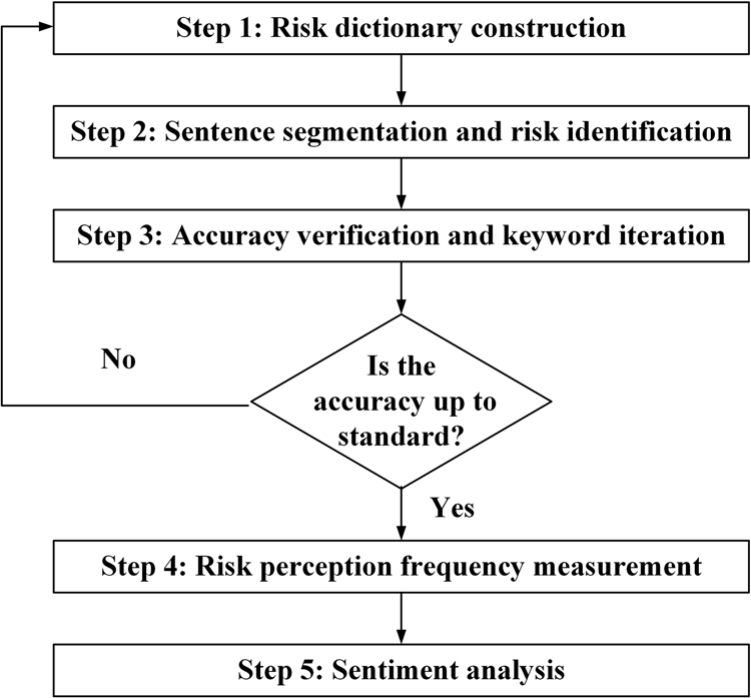
Experimental steps of post-travel perceived risk identification. Identification and measurement process of postravel risk perception.

In addition, in this paper, we do not make a strict distinction between destination image and perceived risks described in the travel notes as mentioned above. However, while perceived risks mainly convey the negative emotions of tourists, the destination image may involve positive perceptions. Therefore, to portray the importance of perceived risks after travel, we resort to sentiment analysis to measure the degree of tourists’ negative perceptions of a risk factor. The experimental process consists of the following five main steps (see Fig. 4).

1. Risk dictionary construction. Based on the final risk lists of the Q&As, as well as prior knowledge of the previewed travel notes, a thematic dictionary was constructed by assigning keywords to each risk type. For example, “altitude stress” and “disease” are keywords related to health risk.
2. Sentence segmentation and risk identification. Travel notes are usually long-winded. To better locate the context for describing risk perception, we used punctuation marks such as periods, exclamation marks, and ellipses as separators to preprocess the travel notes data. Next, following the workflow of the dictionary method, we used the predefined thematic dictionary for risk perception identification at the sentence level. For example, when the words “altitude stress” appear in a sentence, the sentence was considered to describe health risk. It is worth noting that most of the sentences in the travel notes did not contain keywords related to risk perceptions, which means that most of the sentences were useless for risk identification.
3. Accuracy verification and keyword iteration. To ensure the reliability of the experimental results, the research introduced two indicators, “Precision” and “Recall”, to measure the classification accuracy of the model (Li et al., 2022). Among them, Precision measures the percentage of identified sentences belonging to a certain risk type that actually describe the risk, and Recall measures the proportion of sentences describing a certain type of risk that were successfully identified. The Precision and Recall formulas are shown in Eq. (1) and Eq. (2), respectively. If the risk identification results achieve high Precision and Recall, the following step can be initiated. If the results were not satisfactory, the sentences of the travel notes were randomly selected for manual review, and the results guided us to adjust the corresponding set of risk perception keywords. Specifically, if Precision is low, it means that the keywords we predefined are not accurate enough, and some sentences unrelated to corresponding risk perception were included. If the Recall was low, it meant that our keywords were not enough to cover all the sentences discussing the corresponding risks, and we needed to continue to select keywords related to specific risks from the randomly selected sentences.
  1 $${\mathrm{Precision}} = \frac{{{\mathrm{TP}}}}{{{\mathrm{TP}} + {\mathrm{FP}}}}$$
  2 $${\mathrm{Recall}} = \frac{{{\mathrm{TP}}}}{{{\mathrm{TP}} + {\mathrm{FN}}}}$$
4. Risk perception frequency measurement. The previous three steps were repeated until higher Precision and Recall were obtained, and the number of perceptions of each risk topic was counted based on the final thematic dictionary and risk identification results. For example, if a travel note did not have any keywords related to health risk, the sample was considered not to pay attention to health risk. In contrast, if the keywords related to health risks appeared in a travel note, the sample was considered to have perceived health risks. No matter how many times there were related keywords in the same travel note, the entry was counted as a single instance of a risk perception, increasing the count by only 1, indicating that the publisher of the travel note perceived health risks after travel.
5. Sentiment analysis. In this paper, we used five sentiment analysis methods including Long Short-Term Memory (LSTM), Bidirectional Long Short-Term Memory (BILSTM), Convolutional Neural Networks (CNN), Gated Recurrent Unit (GRU), and bag-of-words network (BOW) to calculate the negative sentiment probability of risk perceptions in travel notes. These supervised deep learning methods are more accurate than traditional statistical learning methods. However, supervised deep learning methods require large amounts of labelled data to train available models, which are often difficult to obtain. Fortunately, some open-source projects have pretrained models using a large amount of generically labelled data, among which Baidu’s Sentiment Classification (Senta, https://github.com/baidu/Senta) project has attracted the attention of scholars for its versatility and accuracy. Through training with a large number of self-built corpora, it can automatically determine the emotional polarity of the text and give the corresponding confidence, providing important technical support for understanding user behaviour habits and analysing hot spots. We identify the above five deep learning methods in equations as Senta_LSTM, Senta_BILSTM, Senta_CNN, Senta_GRU, and Senta_BOW.

After sentiment analysis, the negative sentiment probability of risk perception can be expressed by Eq. (3).

3 $${\mathrm{Sent}}\_{\mathrm{Risk}}_i = \frac{{1/M\left( {{\mathrm{Sent}}\_{\mathrm{Risk}}_{i,n,m}} \right)}}{N}$$

where $N$ denotes the number of samples in which ${\mathrm{Risk}}_i$ is mentioned, that is, the perception frequency. $M$ represents the number of sentences that mention ${\mathrm{Risk}}_i$ in the travel note $n{{{\mathrm{ }}}}(n = 1,2,3...N)$.

## Results

### Pre-travel perceived risk identification results

During the experimental process of manually labelling the Q&As, the consistency of risk labelling results between the two participants calculated by Eq. (4) is 0.8903, indicating that the risk labelling results are highly consistent. Among them,${{{\mathrm{N}}}}$denotes the total number of risk categories labelled by the first participant, $S$ denotes the number of all Q&A samples, and $R_i$ denotes the number of risk labels for the ${\mathrm{Sample}}_i$. $B_j^r$ and $A_i^r$ denote the risk labelling results of the second participant and the first participant, respectively. If the second participant’s labelling result for ${\mathrm{Risk}}_j$ of ${\mathrm{Sample}}_i$ is in the set of the first participant’s risk labelling results for ${\mathrm{Sample}}_i$, then $I({{{\mathrm{ }}}})$ takes the value 1; otherwise, it is 0.

4 $${{{\mathrm{Consistency = }}}}\frac{1}{{{N}}}\mathop {\sum}\nolimits_{i = 1}^S {\mathop {\sum}\nolimits_{j = 1}^{R_i} {I\left( {B_j^r\,{\mathrm{in}}\,A_i^r} \right)} }$$

Table 2 presents information on the name, definition, frequency and examples of the 20 risk factors perceived by tourists before travelling to Tibet and whether they are destination-specific. The main types of risks that tourists perceive before travelling to Tibet include travel route selection, traffic, expense, equipment, season selection, and entry procedures (Table 2). These risks, due to their high perceived frequency, may become important factors that hinder tourists’ selection of Tibet, deserving attention from managers. In contrast, tourists have less frequent concerns and perceptions of risks, such as poor communication signals, conflicts with traditional customs, quality of dining and shopping, whether scenic spots are open, and the choice of travel agencies before travel. In addition, since the data were collected in April 2021, there is also relatively little perception of the risk of the COVID-19 epidemic. Among many risks, the features of the Tibet, such as high altitude, harsh climatic conditions, long distances between attractions, weak infrastructure, and widespread religious beliefs, making seasonal risk, entry procedure risk, time risk, climate risk, safety and health risk, infrastructure risk, traditional custom risk, and communication risk are all distinctive characteristics, which tourists need to pay extra attention to before travelling to Tibet.

**Table 2:** Description of pretravel perceived risks in Tibet.

No.	Risk	Definition and example	Count	Frequency	Specificity
1	Route Selection Risk	Uncertainty about the reasonableness of travel route design and choice of attractions, e.g., “I want to ask if the following itinerary is reasonable?”	907	23.27%	
2	Traffic Risk	Uncertainty about transportation, e.g., “Is it better to go to Tibet by plane or train?”	663	17.01%	
3	Expense Risk	Uncertainty about travel costs, e.g., “How much does it cost to travel from Guangzhou to Tibet?”	380	9.75%	
4	Equipment Risk	Uncertainty about equipments, e.g., “What clothes and necessities do I need to prepare?”	304	7.80%	√
5	Season Risk	Uncertainty about whether the travel season is reasonable, e.g., “When is the best time to travel to Tibet?”	300	7.70%	√
6	Entry Procedure Risk	Uncertainty about Tibetan entry procedures, e.g., “Is it necessary to apply for frontier pass?”	204	5.23%	√
7	Time Risk	Uncertainty about the duration of the tour and the schedule of the itinerary, e.g., “How long does it take to go to Tibet from Qinghai?”	188	4.82%	√
8	Climate Risk	Uncertainty about local climatic conditions and concerns about severe weather conditions, e.g., “How strong is the sun in Tibet? What degree of sun protection is required?”	177	4.54%	√
9	Health Risk	Uncertainty about the occurrence of altitude sickness, and concerns about their own health, e.g., “Is the altitude sickness serious in Tibet? How can we avoid it?”	156	4.00%	√
10	Accommodation Risk	Uncertainty about finding suitable accommodation, and concerns about accommodation conditions, e.g., “Is it convenient to get accommodation in Tibet?”	144	3.69%	
11	Security Risk	Uncertainty about the probability of disasters, and concerns about the hazard of accidents, e.g., “I want to go to Tibet on foot with my friends, how can I ensure my safety?”	97	2.49%	√
12	Ticket Risk	Uncertainty about information such as ticket purchases and visit time limits, e.g., “How can I book tickets to the Potala Palace in Tibet?”	92	2.36%	
13	Infrastructure Risk	Uncertainty about the construction conditions of infrastructure such as local road and concerns about its’ convenience, e.g., “How is the road condition?”	80	2.05%	√
14	Travel Agency Selection Risk	Uncertainty about whether the best travel agency and tour group can be selected, and concerns about the quality of travel services, e.g., “I’d like to find a travel agency in Tibet, do you have any good recommendations?”	69	1.77%	
15	Openness Risk	Uncertainty about whether attractions are open, e.g., “Is the Namtso Lake in Tibet open?”	54	1.39%	
16	Dining & Shopping Risk	Uncertainty about the convenience of dining and shopping, and concerns about its’ quality, e.g., “What are the best places for shopping in Tibet?”	33	0.85%	
17	Traditional Custom Risk	Uncertainty about religion customs, as well as concerns about communication barriers and cultural differences, e.g., “Can the locals understand Mandarin?”	25	0.64%	√
18	Epidemic Risk	Uncertainty about pandemic policy, e.g., “Do I need to be quarantined when travelling to Tibet?”	17	0.59%	
19	Communication Risk	Uncertainty and concerns about the quality of communication signals, e.g., “Is there a GPRS signal in most places in Tibet?”	8	0.44%	√
20	Other Risk	Uncertainties and concerns about travel with a partner, jet lag, and other aspects, e.g., “I plan to spend Chinese New Year in Tibet in 2018, is there any companions?”			

### Post-travel perceived risk identification results

**Fig. 5:**
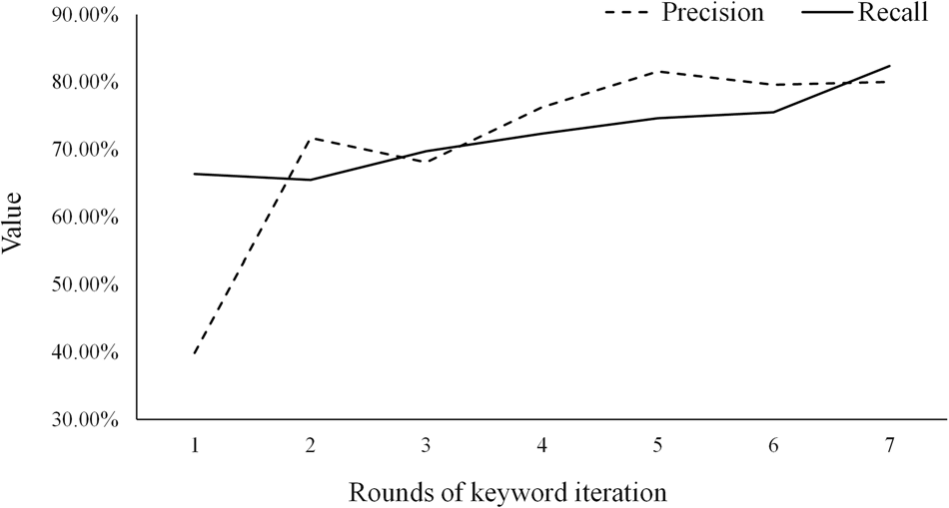
Model accuracy evaluation. The broken line represents Precision and the solid line indicates Recall, and the figure shows the trend of both with the increase of keyword iteration rounds.

In the experimental process of identifying and extracting perceived risks from travel notes, when the keywords were adjusted in the fifth round, the Precision and Recall of the experiment both reached a high and stable state (see Fig. 5). The researchers built a thematic dictionary based on the adjusted keywords in the seventh round to identify tourists’ risk perceptions after travel.

**Fig. 6:**
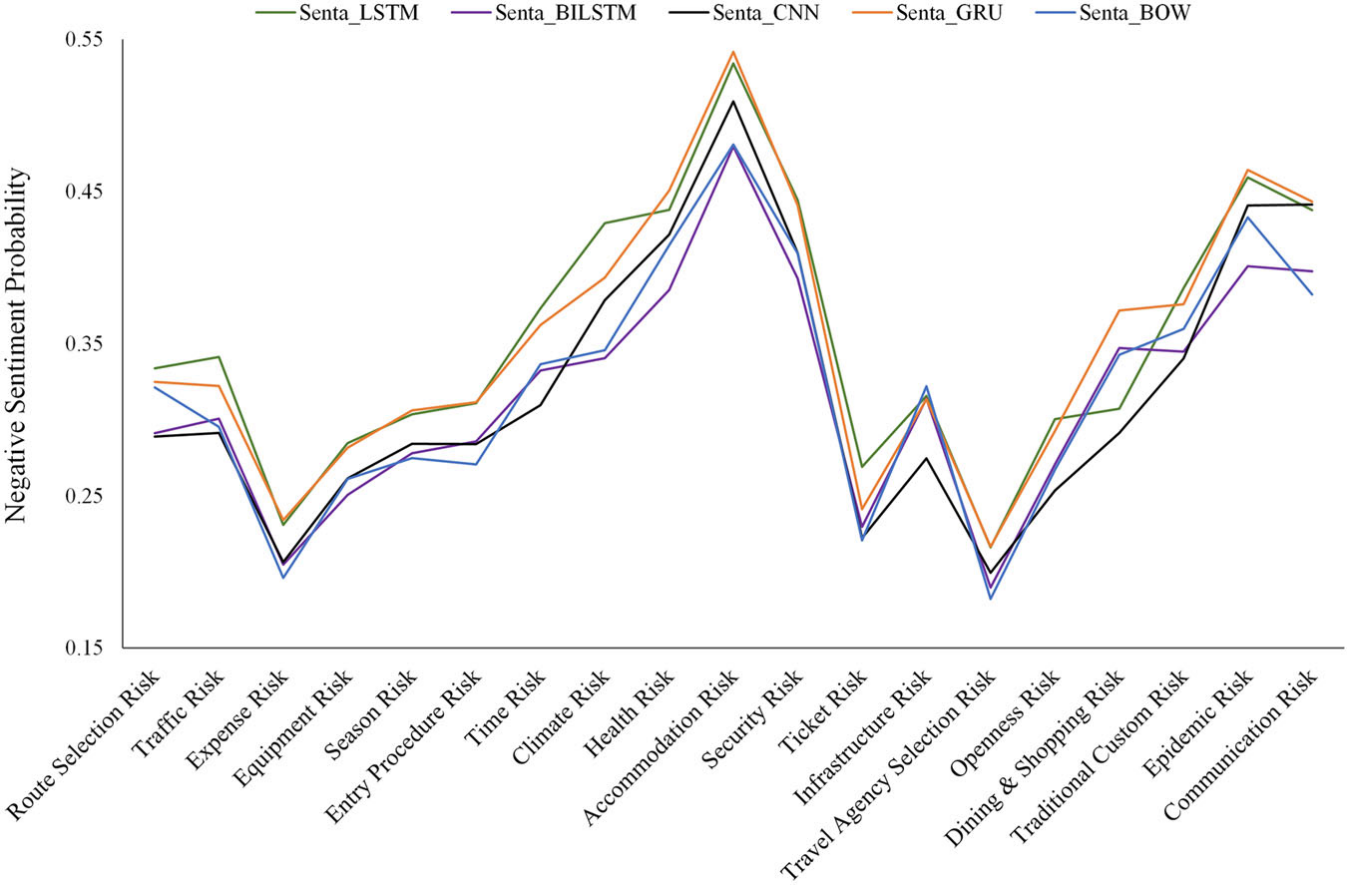
The negative sentiment probability of post-travel perceived risks. Negative sentiment probabilities for each type of risk obtained from the five sentiment analysis methods.

Table 3 and Fig. 6 show the negative sentiment probabilities of tourists’ revised risk perceptions and their trends obtained based on the five sentiment analysis methods (see Table 3 and Fig. 6). The conclusions of the five sentiment analysis methods are relatively consistent, and the robustness of the experimental results is further ensured.

**Table 3:** Negative sentiment probability of post-travel perceived risks.

Risk	Sentiment analysis method				
	Senta_LSTM	Senta_BILSTM	Senta_CNN	Senta_GRU	Senta_BOW
Route Selection Risk	0.29	0.33	0.32	0.32	0.32
Traffic Risk	0.3	0.34	0.32	0.32	0.3
Expense Risk	0.35	0.31	0.37	0.37	0.34
Equipment Risk	0.29	0.31	0.31	0.31	0.27
Season Risk	0.2	0.23	0.23	0.23	0.2
Entry Procedure Risk	0.34	0.43	0.39	0.39	0.35
Time Risk	0.34	0.39	0.38	0.38	0.36
Climate Risk	0.28	0.3	0.31	0.31	0.27
Health Risk	0.39	0.44	0.45	0.45	0.41
Accommodation Risk	0.33	0.37	0.36	0.36	0.34
Security Risk	0.48	0.53	0.54	0.54	0.48
Ticket Risk	0.31	0.32	0.31	0.31	0.32
Infrastructure Risk	0.39	0.44	0.44	0.44	0.41
Travel Agency Selection Risk	0.23	0.27	0.24	0.24	0.22
Openness Risk	0.25	0.28	0.28	0.28	0.26
Dining & Shopping Risk	0.27	0.3	0.29	0.29	0.27
Traditional Custom Risk	0.19	0.22	0.22	0.22	0.18
Epidemic Risk	0.4	0.44	0.44	0.44	0.38
Communication Risk	0.4	0.46	0.46	0.46	0.43

**Fig. 7:**
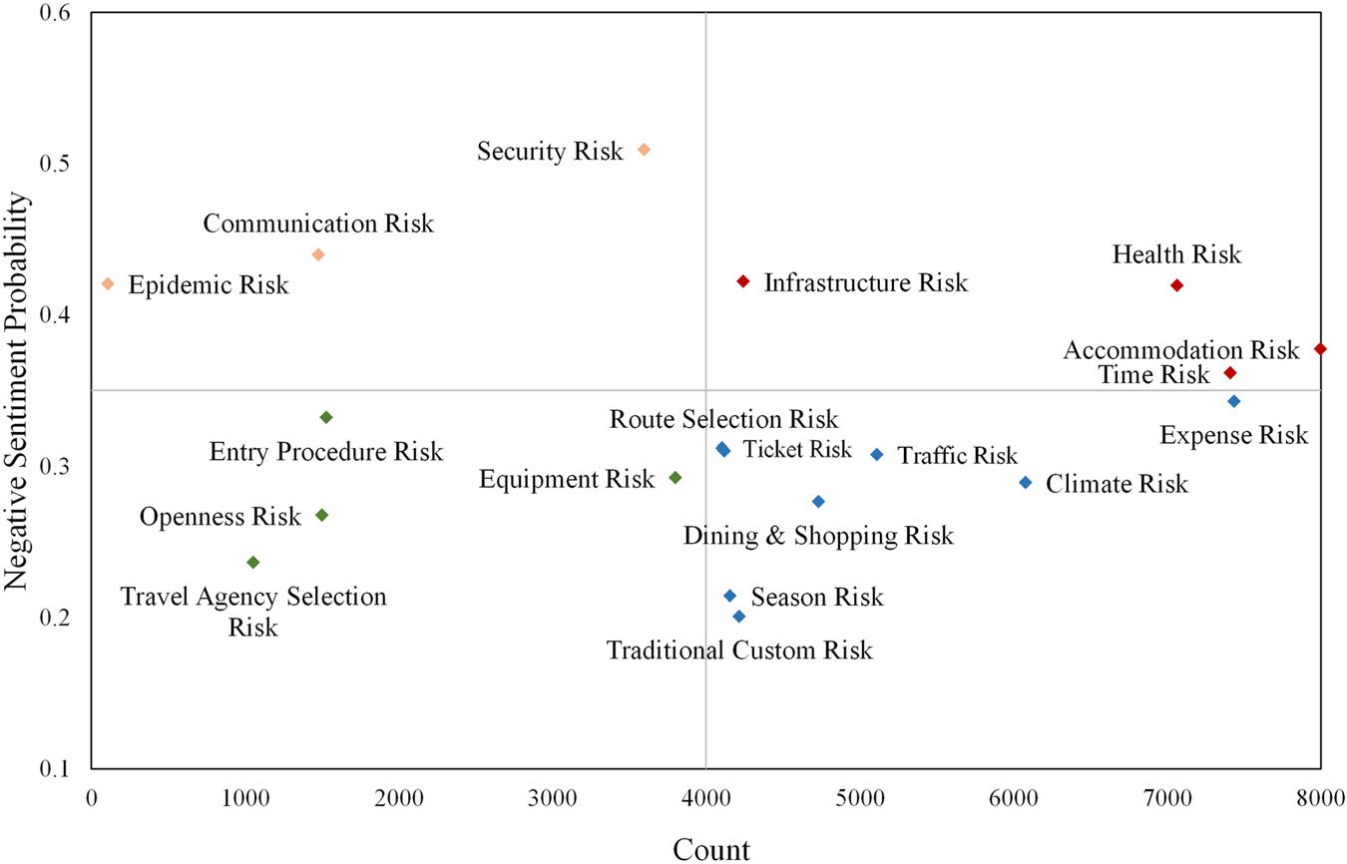
Four-quadrant diagram of post-travel perceived risks. Comparison of risk perception frequency and negative sentiment probabilities after travel.

For further analysis, Fig. 7 depicts a four-quadrant plot regarding the perceived frequency of risks and their average negative sentiment probability based on the five sentiment analysis methods (Fig. 7). As shown in the figure, the perceived frequency and sentiment of the tourists were not consistent across risks after travel. Among them, risks related to infrastructure, health, accommodations, and time were perceived more frequently and had higher negative sentiment probability, and these risk factors may become important influencing factors of tourist destination satisfaction. Risks related to security, communication, and the epidemic belonged to a type in which negative sentiment probability was significantly stronger than perception frequency. These factors are easily ignored by tourists and managers due to their low perception frequency, but once they occur, they can also have serious negative impacts on the destination image. Risks related to tickets, season, transportation, dining & shopping, traditional customs, climate and expense belonged to a type in which the perceived frequency is significantly stronger than the negative sentiment probability, while risks related to entry procedures, openness, equipment and travel agency selection had a low perceived frequency and low negative sentiment probability. Owing to the low probability of negative emotions, the above two types of risks reflected important areas that could be used to enhance the image of the destination.

### Comparison of perceived risks before and after travel

**Fig. 8:**
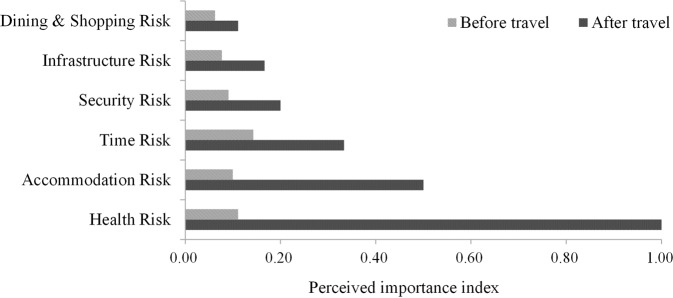
Risks with perceived importance significantly increased after travel. The experiment results indicate that these risks are grossly underestimated by tourists before travel.

**Fig. 9:**
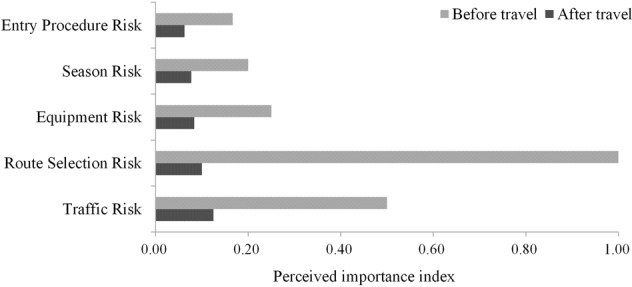
Risks with perceived importance significantly decreased after travel. The experiment results indicate that tourists are overly concerned about these risks before travel.

**Fig. 10:**
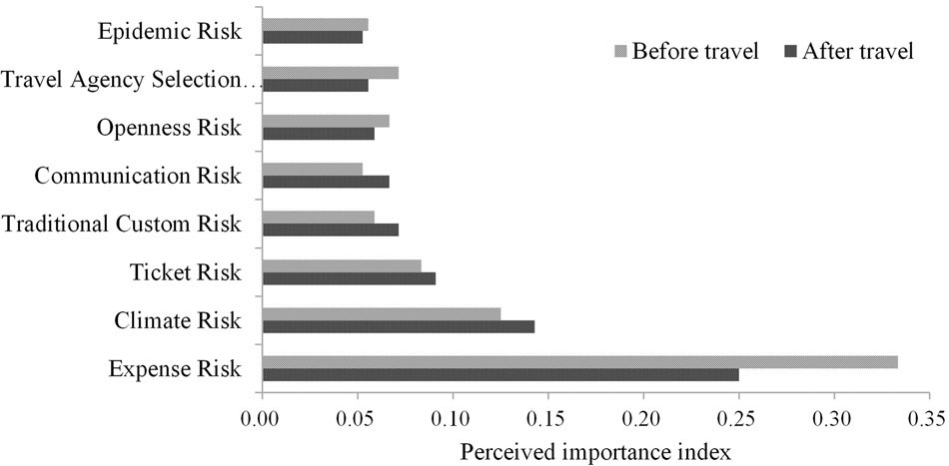
Risks of no significant change in perceived importance before and after travel. The experiment results indicate that tourists’ perception of these risks are relatively consistent before and after travel.

Figures 8–10 show the trends in the perceived importance of different types of risks before and after tourists travel to Tibet. The importance of perceived risks before travel was represented by the perception frequency, and the importance of perceived risks after travel was approximately represented by the frequency of perception multiplied by the probability of negative sentiment, where the negative sentiment probability is the average of the calculation results of the five sentiment analysis methods. To more intuitively reflect the comparative relationship between the perceived risk importance before and after travel, the study ranked the perceived importance of risks before and after travel separately and constructed a perceived importance index by taking the reciprocals. As shown in Figs. 8 to 10, the greater the value of the importance index was, the stronger the perceptions of the risk by tourists. According to the changing trends of the perceived importance of risks before and after travel to Tibet, all perceived risks could be divided into the following three categories: those that are higher after travel, those that are lower after travel, and those that do not change.

Figure 8 illustrates the risks that have a significantly higher perceived importance after travel than before travel (Fig. 8). Risk factors that can be classified into this category mainly included health risk, accommodation risk, time risk, security risk, infrastructure risk, and dining & shopping risk. From the sample of travel notes, tourists’ expressions in relation to these risks were usually relatively negative, such as “In the rainy season, the road conditions are very bad, and unpredictable; after a heavy rain, the roadbed is likely to be washed away, which seriously affects the trip”, “The road conditions are very bad; I only drove 300 kilometres in a whole day”, “Severe high-altitude stress can be fatal”, and “This is the third serious car accident we encountered on this trip, which made us feel that life is unpredictable and fragile. Before we got there, we never felt scared, and never thought that the road conditions would be so bad”.

Figure 9 shows the risks that were perceived to be significantly less important after travel than before travel (Fig. 9). The risk factors that belong to this category mainly include traffic risk, route selection risk, equipment risk, season risk, and entry procedure risk. Specifically, transportation involves the transfer between scenic spots, and the choice of routes is ultimately a trade-off between scenic spots and scenery. Season risk, as defined, are the uncertainties of scenery quality and accessibility in different seasons, while entry procedures are about whether you can go to a scenic spot smoothly. Tourist perception of such risks may be gradually weakened by witnessing the beauty of the local area. For example, “Different seasons have different beauty”, “Now it is very convenient to rent a car and drive by yourself”, and “When I am in a scenic spot, there will be different scenery in different seasons. Summer is green, and autumn is yellow…”.

Figure 10 shows the risks for which the perceived importance did not fluctuate significantly before and after travel (Fig. 10). Except for the above two risk categories that fluctuated significantly, tourists’ perceived importance of most risk factors did not change significantly before and after travel. Among them, expense and climate could be further categorised as risk types that were strongly perceived both before and after travel. In contrast to expense risk and climate risk, traditional custom risk, communication risk, openness risk, travel agency selection risk, and epidemic policy risk were risk types with relatively weak perceptions before and after travel. In addition, although the perceived importance of epidemic policy changes was very low before and after travel, this was mainly due to the limitation of the sample time span. The impact of epidemics and other public health events on tourism activities has been widely verified (Chica et al., 2021; Qiu et al., 2020; Sigala, 2020). During an epidemic, the control of disease risks is undoubtedly a priority for local management (Neuburger and Egger, 2021).

## Discussion

Tourist risk perception plays a key role in the development of the destination tourism market (Chew and Jahari, 2014; Sönmez and Graefe, 1998). Considering that risk perception is highly susceptible to subjective factors such as information acquisition channels, it is necessary to identify the dynamic process of identifying risks before and after travel. This not only guides tourists to form a reasoned assessment of the destination but also provides an important reference basis for destination risk management by government departments. This paper takes Tibet, a tourist destination with a unique risk profile, as a sample to explore tourists’ risk perceptions and their dynamic processes of reassessment after travelling to the destination.

The results prove that there is indeed a significant difference between tourists’ risk perceptions of the destination before and after travel. The findings are consistent with those of Tardivo et al. (2020), Xie et al. (2020), and Zimmermann et al. (2013). In addition, the social amplification of risk framework states that risk events can intensify or attenuate public risk perceptions in interaction with psychological, social and cultural processes (Kasperson et al., 1988). By comparing the risk perception identification results before and after travel, it can be found that the social amplification effect exists in all aspects of tourism activities and has an impact on tourism decisions.

Figure 8 shows that tourists’ perceived importance of the risks closely related to their own health and safety, as well as the completeness of infrastructure such as restaurants and hotels, has increased significantly after travel. It is easy to see from the tourists’ statements that they severely underestimate the severity of these risks before travel, which may be related to optimism bias. Optimism bias refers to people’s tendency to believe that they are less susceptible to a disease or other negative outcomes than others, and people usually expect positive things to happen in the future even though there is no reason to make this assumption (Weinstein, 1989). For example, people tend to believe that they are less likely to be victims of car accidents or earthquakes and less likely to experience illness or depression than others (Helweg-Larsen and Shepperd, 2001). Therefore, when travelling to Tibet, even in the face of high-altitude stressors and poorly maintained road conditions, tourists may still underestimate their risk susceptibility and the risk severity due to optimism bias. However, overly optimistic psychological expectations may lead to lower tourist satisfaction (Kwon and Lee, 2020; Wilson et al., 2005). Figure 9 shows that after travelling, tourists significantly reduce the perceived importance of risks directly related to attractions and scenery, such as season, travel route, and transport. Given the high perceived importance of such risks prior to travel, they may act as key constraints preventing potential tourists from travelling to Tibet.

Intensified or attenuated risks will trigger corresponding behavioural responses, which in turn act as an “amplification station” to have a secondary effect on other tourists’ risk perceptions (Renn et al., 1992). Unlike experts who judge risk based on research results and statistical evidence, laypeople rely mainly on personal intuition to assess risk from limited information such as media reports (Slovic, 1987). Therefore, for a better tourism experience and economic development at the destination, tourists and destination managers need to conduct two-way risk communication (Matta, 2020), which can guide tourists to form more accurate risk assessments and guide managers to optimise destination risk management. For risks whose perceived importance increases significantly after travel, managers need to strengthen control over these liabilities and raise tourist awareness of risk prevention. Proper management can reduce the severity of altitude sickness and the probability of car accidents, thus increasing destination satisfaction. For risks whose perceived importance decreases after travel, efforts need to be invested in public awareness to reduce anxiety and thus attract more tourists.

In terms of communication methods, previous studies have shown that numerical risk communication, such as a risk occurrence probability, has difficulty conveying effective and accurate perceptions. In contrast, posters, graphs, and announcements are more capable of conveying risk information, thus enhancing tourists’ risk perceptions (Witte and Allen, 2000). Therefore, diversified forms of information communication are expected to make greater contributions to accurate assessment of destination risks. Although the media has a nonnegligible role in the social communication of risk (Kapuściński and Richards, 2016), the general public is also a particularly important disseminator of risk information (Kusumi et al., 2017). Therefore, tourists who fully read online reviews before travelling and take advantage of interpersonal communication are aided in forming a more accurate risk perception.

Figure 10 shows that some risks have relatively consistent perceived importance before and after travel. Management of risk factors that are strongly perceived are expected to be an effective way to attract more tourists. Therefore, for expense risk, which is controllable, managers should focus on optimising the local tourism fee structure and creating a favourable business environment. For climate, which cannot be manually controlled, popular science dissemination should be strengthened to guide tourists to form correct expectations and make perfect risk plans. Risk factors that are perceived weakly will not become key factors affecting tourists’ travel decisions but may be additional options to enhance tourism satisfaction. Therefore, increasing cultural promotion, promoting ethnic and cultural integration, and speeding up the work of laying infrastructure, such as communication networks, may reap more positive feedback.

### Strengths and limitations

In this paper, we address the problems in dynamic risk perception identification from both data analysis and critique of existing techniques. We portray the change in tourists’ risk perceptions after travelling to Tibet. Despite the generality of the research paradigm, considering the unique economic, cultural and geographical conditions of the Tibet, the identification results for risk perception and its changing patterns in this paper may not necessarily be generalised to other regions. Another limitation is that due to the strict travel restrictions imposed in China after the COVID-19 epidemic, there was a period of missing data, making tourist perception of the epidemic risk poorly represented. Future research needs to consider more detailed information, for example, taking gender into consideration to further explore the differences in risk perception trends between male and female tourists, to provide more evidence for accurate tourism risk management.

## Conclusions

To explore an effective identification programme for tourists’ dynamic risk perceptions, we introduced a research framework based on Q&As and travel notes, as well as text mining technologies, and took Tibet as an example to portray tourists’ risk perceptions existed before and after travelling there.

The results show that there are significant differences in tourists’ risk perceptions of the destination before and after travel. Specifically, tourists’ perceived importance of health and safety risks increased significantly after travel, while the perceived importance of risks directly related to attractions and scenery showed a downwards trend. In addition, expense and climate are strongly perceived as risks before and after travel, while risks associated with traditional customs, communication, openness, and travel agency selection are relatively weakly perceived both before and after travel. Therefore, to attract more tourists and enhance their travel experience, it is necessary to understand the dynamic change in tourists’ risk perceptions. Local tourism management departments should tailor their destination risk management and conduct effective risk communication to guide tourists to form accurate risk perceptions.

## Data Availability

The authors declare that all the data reported in the current article are publicly available from the Ctrip (https://www.ctrip.com/). The code scripts are available from the corresponding author upon request.
